# Biodegradable Conducting Polymer-Based Composites for Biomedical Applications—A Review

**DOI:** 10.3390/polym16111533

**Published:** 2024-05-29

**Authors:** Tabrej Khan, Gayathri Vadivel, Balan Ramasamy, Gowtham Murugesan, Tamer A. Sebaey

**Affiliations:** 1 Department of Engineering Management, College of Engineering, Prince Sultan University, Riyadh 11586, Saudi Arabia; 2 Department of Physics, KPR Institute of Engineering and Technology, Coimbatore 641407, Tamil Nadu, India; 3 Department of Physics, Government Arts and Science College, Mettupalayam 641104, Tamil Nadu, India; 4 Department of Physics, Kongunadu Arts and Science College, Coimbatore 641029, Tamil Nadu, India; 5 Department of Mechanical Design and Production Engineering, Faculty of Engineering, Zagazig University, Zagazig 44519, Sharkia, Egypt

**Keywords:** conducting polymers, biodegradable polymers, tissue engineering, biomaterials, antibacterial

## Abstract

In recent years, researchers have increasingly directed their focus toward the biomedical field, driven by the goal of engineering polymer systems that possess a unique combination of both electrical conductivity and biodegradability. This convergence of properties holds significant promise, as it addresses a fundamental requirement for biomedical applications: compatibility with biological environments. These polymer systems are viewed as auspicious biomaterials, precisely because they meet this critical criterion. Beyond their biodegradability, these materials offer a range of advantageous characteristics. Their exceptional processability enables facile fabrication into various forms, and their chemical stability ensures reliability in diverse physiological conditions. Moreover, their low production costs make them economically viable options for large-scale applications. Notably, their intrinsic electrical conductivity further distinguishes them, opening up possibilities for applications that demand such functionality. As the focus of this review, a survey into the use of biodegradable conducting polymers in tissue engineering, biomedical implants, and antibacterial applications is conducted.

## 1. Introduction

Biomaterials, whether organic or manmade, can degrade naturally and positively interact with living things [1] and play a pivotal role in the biomedical field. These materials, precisely characterized as a class intended to interact with biological systems to diagnose, treat, and substitute physical organs, tissues, or functions [2,3], are a crucial element within humans’ environment-based or natural health system [4]. Among these biomaterials, biopolymers are the key category thanks to their biocompatibility, biodegradability, and safety for human health [5]. Categorized by their characteristics, these biopolymers are divided into groups that distinguish between biodegradable and non-biodegradable types [6,7].

Biodegradable polymers stand out as the preferred choice in biomedical applications, given their remarkable biocompatibility. The discipline of biomedical engineering is an appealing one, since it combines engineering and biology to develop basic engineering components and materials for the healthcare and medical industries. By combining cells, scaffolds, and bioactive chemicals, tissue engineering aims to replace or recover ailing or damaged body tissue. Scaffolds should integrate several features, including biodegradability, biocompatibility, the appropriate level of mechanical strength, sufficient porosity for the transit of tiny molecules, controllability, implantation, and sterilizing. In the production of scaffolds, excellent choices contain synthetic aliphatic polyesters and natural polymers like chitosan and gelatin. However, the absence of sites for covalent bonding and poor hydrophilicity limit the cells’ ability to adhere to surfaces. Although biopolymers and ordinary polymers are biodegradable, they are natural insulators, which restricts their usage in several biological applications where biomaterial conductivity is required [8]. Materials demonstrating enduring and dependable performance are essential for various implant applications within the body, encompassing dentistry, bone replacement, and bone fixation. Beyond biodegradability, biomaterials must possess various qualities to be suitable for applications such as gene therapy, tissue engineering, regenerative medicine, and controlled drug administration [9,10,11].

Since the early 20th century, various biomaterials, including synthetic polymers, composites, ceramics, alloys, metals, and carbons, have been extensively used [12,13]. These novel biomaterials showed superior mechanical qualities, repeatability, and enhanced biological and chemical functioning. Biomaterials, emerging materials that interact with biological components to facilitate medical diagnosis and treatment, find diverse applications. These include biosensors, artificial muscles, drug delivery, tissue engineering, and other subjects [14].

**Figure 1:**
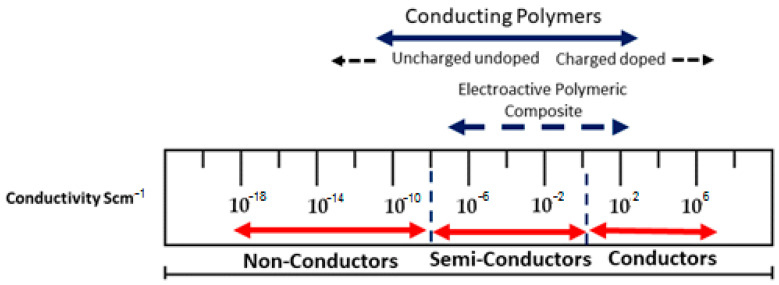
The spectrum of conductivity observed in conducting polymers and conductive polymeric composites.

Since the discovery of intrinsically conducting polymers, researchers have explored their unique electronic properties, including their low-energy optical transmission, low ionization potential, and high electron affinities. These properties make them suitable for various applications such as thin-film transistors, organic light-emitting diodes, sensors, supercapacitors, organic solar cells, and electrochromic displays. Numerous research groups have extensively investigated conducting polymers for these applications, with several excellent reviews available. With extensive research in this area, conducting polymers and electroactive polymers are now garnering attention for their potential biomedical and ani-bacterial applications. These materials find extensive use in various fields, such as skin tissue engineering, drug delivery, orthopedics, dentistry, and cardiovascular devices. They are crafted to seamlessly interact with biological systems, aiming to replace, assess, or repair damaged or compromised bodily tissues, organs, or systems [15], in addition to their roles in tissue engineering, biosensors, artificial muscles, and medication administration [16,17,18]. The conductivity range depicted in Figure 1 illustrates the spectrum of conductivity observed in conducting polymers and conductive polymeric composites.

Polymer composites comprise two components: filler and a polymer matrix. Conductive polymer composites consist of a polymer matrix combined with conductive filler, which can be either intrinsically conductive or inorganically conductive.

**Figure 2:**
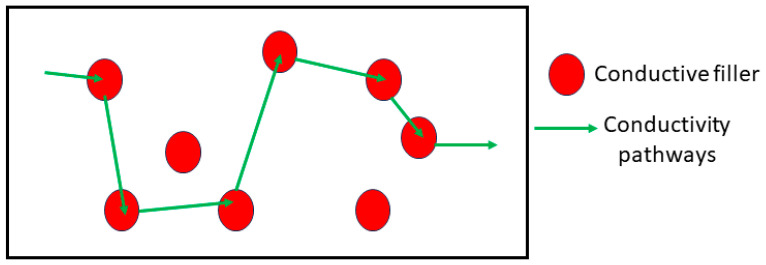
Schematic diagram illustrating potential conductivity pathways in composite polymers.

The polymer matrix contributes to the mechanical and physical qualities, and the conductive fillers contribute to the electrical properties. The volume content of the filler significantly influences the conductivity of the composites. Figure 2 depicts a schematic diagram of potential conductivity channels in composite polymers. When conductive particles form channels adjacent to one another, electricity can flow along these conductivity pathways.

Researchers are keenly interested in advancing polymer systems that possess both biodegradability and electrical conductivity within the biomedical field. Such systems are regarded as promising biomaterials due to their essential qualities of electrical and biological properties, which are crucial prerequisites for biomedical applications. In addition to being biodegradable, these materials also showed certain excellent qualities that made them useful in various engineering domains [19,20,21,22], including good processability, chemical stability, cheap production costs, conductivity, etc. We shall discuss these kinds of biodegradable conducting polymers in the current review discussion of tissue engineering, biomedical implants, and antibacterial applications. Biomaterials have gained significance in contemporary society, driven by advancements in material processing and medicine over the past several decades.

## 2. Biodegradable Materials

**Figure 3:**
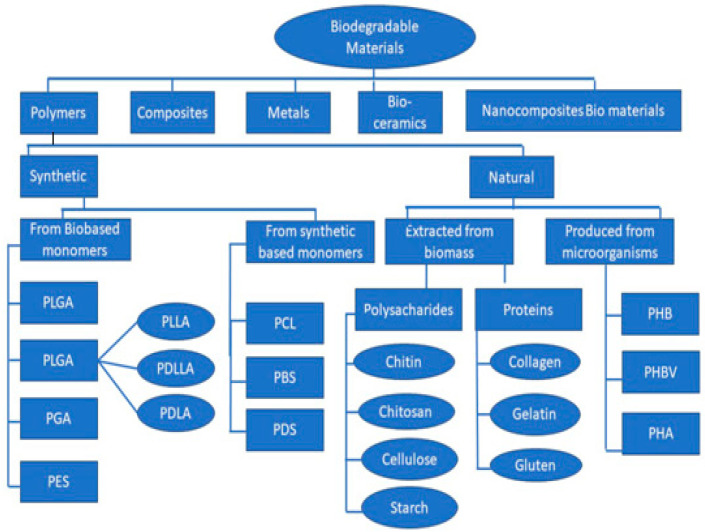
Primary and secondary groups of biomaterials and the categorization of biodegradable polymeric biomaterials based on their source and processing technique.

Advancements in tissue engineering, regenerative medicine, gene therapy, and controlled drug delivery have amplified the demand for enhanced biomaterials. A comprehensive understanding of biodegradability is crucial, particularly concerning polymers, and novel materials must be tailored to possess precisely controlled degradability [23,24]. We recommend organizing them into five primary categories, and it is advantageous to classify both subgroups of polymeric biomaterials, namely, natural and synthetic, into classes and subclasses (Figure 3).

This approach offers various benefits, such as streamlining the selection process for specific applications. By controlling cell phenotypic expression, the natural extracellular matrix (ECM) can coordinate stromal cells in synthesizing new tissue, especially in response to injury. As an artificial ECM within a scaffold, biomaterials must possess mechanical and biological characteristics compatible with natural bodily tissues. They play a crucial role in establishing a two- or three-dimensional environment that facilitates the growth of new tissues with the correct structure and function. Additionally, they assist in localizing and transporting cells and transforming factors to specific locations within the body. As a result, the design and selection of biomaterials are essential, aiming to control the shape and function in a predetermined manner for the production of engineered tissues [25].

One crucial characteristic among these is that the release of breakdown products can be effectively eliminated from the body through metabolic pathways without causing inflammation. According to the American Standard Testing Materials (ASTM), degradation is “plastic designed to undergo a significant change in chemical structure under specific environmental conditions, resulting in a loss of some properties and its applications in a certain period.” Therefore, it is crucial to monitor the concentration of degradation products in tissues and control the rate of deterioration [26]. Various degradation processes, including enzymatic, hydrolytic, and biodegradation, need consideration [27]. Additionally, it is imperative to account for abiotic processes such as photodegradation, oxidation, and hydrolysis. Depending on environmental factors, these processes may influence how a polymer breaks down before or during the degradation process [28].

There are instances where the term “biodegradation” is inaccurately attributed [29]. For example, though an enzyme may break down a material in vitro, the absence of necessary body enzymes in vivo may impede the degradation process. Hence, cell activity is essential for biodegradation. In contrast, hydrolysis or hydrolytic degradation, rather than biodegradation, is more fitting for the breakdown occurring in vivo due to water-mediated hydrolysis in tissues and organs. Technically, biodegradation refers to the breakdown of a substance due to specific biological activity with an identified mechanism. This is akin to a cell-mediated chemical modification, where bio-alteration—strictly speaking, not biodegradation—is the primary chain scission event.

## 3. Biodegradable Polymers

The literature reported earlier has examined the use of biodegradable polymers as biomaterials [30]. To be considered biomaterials, these polymers must possess three essential characteristics: mechanical resistance, biocompatibility, and bio absorbability. Biodegradable polyesters, known for their adjustable breakdown rates and overall acceptable strength, are commonly utilized in tissue engineering for creating porous structures [31,32]. These studies comprehensively detail these polymers’ chemical composition, breakdown products, breakdown processes, mechanical properties, and therapeutic limitations. Furthermore, biodegradable polymers find applications as implantable matrices or absorbable sutures, facilitating controlled drug release within the body. A previous compilation listing various commercially available biodegradable medical products and their respective uses can be found in [33].

Polymeric materials have a long history of use in clinical applications [34,35,36]. However, depending on the intended use, diverse biological and clinical requirements come into play. Various processes have been employed to develop and modify a range of formulations tailored to meet specific needs for therapeutic applications. This process usually involves controlling factors such as molecular weight, polydispersity, crystallinity, thermal transition, and degradation rate. Collectively, these factors significantly influence the properties of the polymer scaffold [37].

### Classification of Biodegradable Polymers

Biodegradable polymers can be classified into three main categories: natural, synthetic, and hybrid materials. Both natural and synthetic sources can undergo degradation, though synthetic biodegradable polymers are more commonly employed in medical settings. This is likely because they are more chemically modifiable and allow for the creation of customized designs [38]. Recently, hybrid materials have gained attention due to their superior properties in regenerative medicine. Utilizing assembly techniques allows for fabricating these materials with high structural precision, enabling control over properties such as porosity, stiffness, and degradation [39]. Composite or hybrid materials have found extensive use in clinical applications due to their behavior that enhances qualities for scaffold function [40,41,42]. Blending synthetic with natural, natural with synthetic, and synthetic with synthetic polymers has yielded polymeric material blends showcasing improved cell compatibility, processability, and mechanical properties [43]. Incorporating bioactive phases in ceramics enhances the hydrophilicity and water absorption of the polymer matrix by facilitating the rapid exchange of protons in water. This, in turn, influences the degradation process of polymers [44]. Using biodegradable hybrid materials offers several potential advantages, including improved conditions for cell seeding, survival, growth, and differentiation. This is attributed to the osteoconductive function provided by bio ceramics, enhancing crucial mechanical properties for load-bearing applications [45]. Hybrid materials enable the creation of organ-specific tissue-engineering constructs, offering a range of tunable physicochemical properties due to their composition, structural flexibility, and functional adaptability.

The extracellular matrix (ECM) forms an organized network comprising various proteins and polysaccharides, offering structural support to cells. Polymers derived from nature possess the capacity to enhance cell adhesion and activity. Scaffold construction commonly relies on natural polymers such as collagen, gelatin, silk, and alginate [46,47]. Concerns about immunogenicity, pathogen transmission, and the intricate structural composition of natural polymers have driven the development of synthetic polymers as scaffolding materials. Synthetic polymers offer easier processing and a more regulated structure, and they typically pose less immunological risk [48]. This overview outlines the most widely used synthetic polymers in tissue-engineering applications.

In in vitro tissue culture, aliphatic polyester polymers can be amalgamated to produce durable, porous materials that are prefabricated 3D scaffolds. These materials exhibit stability and resist melting or disintegration. Aliphatic polyesters comprise this class, a category encompassing the synthetic biodegradable polymers most employed in tissue regeneration [49]. The ester groups within the backbone of these polymers are typically hydrolyzed, and the rate and nature of degradation products can be adjusted based on the polymer’s molecular weight, content, and structure [50]. Polyanhydrides, easily manufactured from readily available and inexpensive materials, can be tailored to possess desired properties [51]. In vivo, polyanhydrides are both biocompatible and biodegradable, producing harmless diacid byproducts that the body can excrete as metabolites. Initially designed for drug delivery applications due to their pronounced hydrophobicity, these polymers have found various applications. When encapsulated in these polymers, drugs can be effectively shielded, since minimal water infiltration occurs until the polymer undergoes erosion [52,53]. These polymers have also been investigated for use in scaffolds for tissue engineering.

Polyurethanes (PUs) continue to be extensively employed in various biomedical applications, remaining one of the most widely used biomaterial classes. Their popularity is attributed to their segmented-block structural characteristics, which provide a broad range of plasticity with adjustable mechanical, physical, and biological properties. Additionally, they exhibit compatibility with blood and tissues and, more recently, biodegradability [54,55,56,57,58]. Table 1 provides a summary of polymers and their significance in different applications.

## 4. Applications of Biodegradable Conducting Polymer Based Composites

### 4.1. Tissue Engineering

Tissue engineering is an interdisciplinary field that combines principles from engineering and biology to create biological substitutes to enhance, maintain, or repair tissue function. Tissue-engineering applications necessitate the utilization of biodegradable scaffolds. Biodegradability is required to prevent the need for implant removal surgery and to prevent a persistent and lifelong immune response. Key characteristics of a scaffolding biomaterial are biodegradability, biocompatibility, and considerable processing flexibility [78,79]. Electrical conductivity within three-dimensional scaffolds is crucial for enhancing tissue repair and regeneration, particularly in electroactive tissues like cardiac and neural tissues. Chemical, mechanical, and electrical properties must be combined to create scaffolds for tissue regeneration, cell adhesion, and cell proliferation. The conductivities of many tissues, including skeletal muscle, lung, heart, and nerve tissues, vary between 0.03 and 0.6 S/cm. The surface characteristics of biomaterials significantly influence how a cell interacts with the altered substrate and its functioning. Therefore, retaining cells on the material’s surface instead of within their scaffolds proves challenging. Conducting materials can be employed [80,81,82]. Rivers et al. (2002) [83] represent an innovative approach in tissue engineering by developing a conducting biodegradable polymer. This polymer was created by incorporating conducting oligomers of thiophene and pyrrole, which are conductive organic compounds with degradable ester linkages. This polymer exhibits a close interaction with tissues and cells on biological and electrical levels. These kind of polymers were ideal for tissue-engineering applications, since they functioned fantastically to promote tissue regeneration by acting as a transient scaffold for cell adhesion and producing electric signals. Schmidt and Rivers (2004) [84] innovatively designed biodegradable conducting polymers by incorporating aliphatic chain ester linkages with conducting polymers like pyrrole and thiophene. These modified polymers make them valuable tools for addressing various biomedical challenges, ranging from bone repair and muscle regeneration to nerve tissue engineering and wound healing. According to Huang et al. (2007) [85], the condensation process between aniline pentamer and polylactide resulted in the formation of PLA-b-AP-b-PLA, a biodegradable and electrically active triblock copolymer. The conductivity of the two-block components in the PLA-b-AP-b-PLA was discovered to be 5 × 10^−6^ S/cm, because they were microphase separated. Due to its outstanding solubility in most organic solvents, biodegradability, biocompatibility, and ease of processing, this material can be employed as a scaffold in tissue engineering for the heart or brain. Subramanian et al. (2012) [86] reported that poly (3-hexylthiophene) (PHT) and poly (lactideco-glycolide) (PLGA) were modified to create electrically conductive nanofibers. These aligned nanofibers were studied in vitro to see how they affected the adherence and growth of Schwann cells. To create a scaffold for neural regeneration, PLGA-PHT fibers were used. Baheiraei et al. (2014) [87] developed an electroactive polymer composed of polyurethane and aniline pentamer to explore the impact of electrical stimuli on cellular activity, particularly for applications in tissue engineering. In bone tissue engineering, Xie et al. (2015) [88] engineered an aniline trimer/star-shaped polylactide polymer network with robust mechanical properties, excellent shape memory, electroactivity, and adjustable degradability. Moreover, this electroactive network demonstrated superior efficacy in promoting osteogenic differentiation in C2C12 cells compared to polylactide, significantly enhancing their proliferation rate. Song et al. (2016) [89] observed that adding hydroxyapatite to the graphene oxide/polypyrrole composite structure promoted the proliferation of MC3T3-E1 cells compared to the control group without hydroxyapatite. As reported by Yang et al. (2016) [90], the conductive hybrid hydrogel composite, comprising PPy and alginate, serves as an intelligent interface capable of stimulating stem cells through electrical and mechanical impulses. This makes it a promising scaffold for the development of multifunctional brain tissue. Results indicated that hMSCs assumed larger and more elongated forms when various substrates were employed, indicating successful interaction with the conductive hybrid hydrogels. In a study by Silva and colleagues (2018) [91], PEDOT-co-PDLLA emerged as a novel biodegradable and electrically active copolymer synthesized using persulfate as an oxidant. Charge density characteristics, surface chemistry, conductivity, cytotoxicity, and in vitro biodegradability investigations demonstrated its considerable potential for use as scaffold material in various applications, including brain tissue engineering. Xu et al. (2018) [92] introduced a conductive composite scaffold comprising PEDOT as a conductive polymer layer and carboxymethyl chitosan as a biodegradable component. The findings indicated that the composite scaffold supported cell adhesion, vitality, and proliferation and exhibited cytotoxin-free characteristics. Meanwhile, conductive layers maintained biocompatibility in brain tissue engineering while improving mechanical strength and conductivity. Borah et al. (2020) [93] created a nanocomposite by combining chitosan with polyaniline, showing potential for various biological applications. Glycine N-hydroxysuccinimide (NHS) ester was used to modify the surface of PANI/chitosan nanocomposites to generate bioactivity for enhanced cell–biomaterial interactions. As per SEM and confocal microscopy, this surface modification enhances fibroblast cell adhesion, morphology, proliferation, and spreading, highlighting the significance of nanocomposites in tissue-engineering applications. According to Ali Maziz et al. (2021) [94], the brittle backbone and non-degradable nature of conducting polymers may result in poor mechanical properties. Various methods have been documented to overcome these limitations in biological applications when creating their composites.

### 4.2. Biomedical Implants

Polymers are crucial in numerous medical applications, encompassing wound healing, drug administration, and tissue replacement. To maintain the integrity of tissue form and structure and to safeguard cells and damaged tissue, scaffolds are employed during implant placement to support the defective sections temporarily. To assist in the healing and regeneration of injured tissue, the scaffold must have suitable mechanical qualities that match the corresponding defective components. Therefore, sufficient mechanical support is crucial for tissue-engineering applications. Similarly, the scaffold material must ensure benign biocompatibility, bioactivity, no harmful side effects, no stimulation, and no inflammation. This assurance is crucial, as the material will be implanted into human bodies and come into contact with injured tissue [57,58,59]. The scaffold can fulfill its intended role only when transplanted into the human body. Boutry et al. (2010) [95] utilized PLLA-PPy and PCL-PPy composites to construct an RLC resonator circuit through an emulsion polymerization technique. This approach could be employed to develop bioelectronic devices and biodegradable biomedical implants. Lu et al. (2010) [96] prepared hybrid organic–inorganic composites composed of polypyrrole (PPy) and carbon nanotubes, which were effective as electrodes for nerve probes implanted into patients long-term. Wan et al. (2014) [97] discovered that PLLA showed enhanced compatibility with the inclusion of Mg and MgFl_2_, which has great potential in orthopedic implant applications. Ghaziof et al. (2017) [98] employed solvent casting and vacuum drying to create a multi-walled carbon nanotube/polycaprolactone scaffold. They discovered that including 1 wt% MWCNT improved the composite scaffold’s electrical conductivity and mechanical behavior compared to a pure PCL scaffold, and it might be used as a suitable construct for myocardial tissue regeneration. Iron oxide (Fe_3_O_4_) nanoparticles were incorporated into a PLLA bone screw by Wang et al. (2017) [99]. The study demonstrated that the nanocomposite bone screw exhibited satisfactory mechanical properties and did not adversely affect rabbit bone tissue. As a result, the nano-Fe_3_O_4_/PLLA composite was shown to be suitable for use as an implant. Loffler et al. (2017) [100] and Chen et al. (2018) [101] reported that the electrical connection of implanted devices can be accomplished using conducting polymers. These materials offer far more potential than interface materials utilized in traditional metallic implants because of their inherent nature and qualities that resemble biological tissue. Though these biopolymer implants are not yet clinically suitable, Anh-Vu Do et al. (2018) [102] discovered that their high porosity and porous structure significantly reduce their mechanical strength. To tackle this issue, extensive research has been conducted to enhance the mechanical properties of polymers. It has been observed that low-dimensional nanomaterials, such as metal oxide nanoparticles, can serve as a viable and efficient reinforcement to improve mechanical properties. Eslami et al. (2018) [103] found that a poly (lactic-co-glycolic acid) (PLGA) scaffold’s strength, compressive modulus, and biological activity were all increased by the inclusion of titanium dioxide (TiO_2_) nanotubes. TiO_2_ nanotube reinforcement improved osteoblast cell spreading, which promoted cell attachment. Compared to a pure PLGA scaffold, enhanced bone growth around the implants was observed. Electroconductive hydrogels, consisting of a poly (HEMA)-based hydrogel component and a PPy conductive polymer network, have been utilized for surface modification of long-term implanted neurological electrodes and prosthetic devices due to their notable mechanical strength and low cytotoxicity (Kenry and Liu, 2018) [104]. Barua et al. (2019) [105] incorporated zinc oxide (ZnO) nanoparticles into HAP/poly (methyl methacrylate) (PMMA) using gas foaming to create porous scaffolds. When compared to the HAP/PMMA scaffold, the ZnO/HAP/PMMA scaffold exhibited a 143.3 percent increase in elastic modulus and a 252.2 percent improvement in tensile strength.

As indicated by Wu et al. (2019) [106], the chemical biofunctionalization of biomolecules may contribute to the increased structural stability and biocompatibility of conducting polymer films in biological environments. This enhancement could improve the bio interface of implantable devices. Gupta et al. (2019) [107] reported that implantable devices of electrically conductive polymers can produce pacemakers, measure cardiac activity, deliver electrical impulses, etc. Jayaram et al. (2019) [108] performed a detailed investigation on the influence of the addition of MWCNT on the electrical and morphological characteristics of PEDOT: PSS scaffolds. The MWCNT-modified scaffolds showed strong cytocompatibility and biofunctionalization capabilities, crucial for biosensing and cell adhesion, and increased electrical conductivity. These high-performance systems can prove beneficial in applications such as tissue stimulation and electronic implants, where electrical performance is particularly important. Mohammadalizadeh et al. (2020) [109] developed a scaffold by blending a polyhydroxybutyrate/chitosan solution with varying weight percentages of multi-walled carbon nanotubes. They observed an increase in tensile strength by adding one weight percent CNT. Moreover, polyhydroxybutyrate/chitosan/MWCNT scaffolds enhance cell adhesion and growth for cartilage tissue-engineering applications, as indicated by a study on a chondrocyte cell culture. According to Guo et al. (2020) [110], adding inorganic nanoparticles (such as TiO_2_, ZrO_2_, and Al_2_O_3_) to the Ppy coating may improve its mechanical characteristics and corrosion resistance. Hence, synthesizing a multifunctional Ppy/ZnO composite coating for orthopedic implants could prove advantageous. Pei Feng et al. (2021) [111] reported using biopolymers in creating safe, non-toxic implants that exhibit a high level of biocompatibility and can dissolve into carbon dioxide and water within the human body. These biopolymers can be processed into various forms and possess specific mechanical strength, making them extensively studied in tissue defect healing. Bone scaffolds, cartilage tissue scaffolds, bone screws, vascular scaffolds, and other biopolymer implants are often employed. For example, various biopolymer materials are being used to fabricate three-dimensional porous scaffolds, such as PLLA, PGA, PCL, and PHB scaffolds, from bone tissue and cartilage tissue. Jiang et al. (2021) [112] used electrospinning to develop a vascular scaffold by incorporating CNT into PCL/Gelatin. The scaffold exhibited favorable mechanical properties comparable to native vessel characteristics, and the researchers observed its ability to promote the growth of endothelial cells.

Polymers play a crucial role in 3D-printing manufacturing due to their excellent processability, adaptability, and compatibility with various additive manufacturing (AM) methods [113]. Key features include improved strength, temperature resistance, accuracy, surface details, and high precision [114]. Polymers constitute 51% of polymer parts, 29% of metal and polymer combinations, and 19.8% of metal products in additive manufacturing [115]. Reactive monomers, thermoplastic filaments, powder, and resin are frequently employed polymer types in additive manufacturing (AM) processes [116]. Despite the variety of available AM techniques, improvements in polymer printing primarily revolve around four main strategies: (1) powder bed fusion processes, such as selective laser sintering (SLS); (2) deposition-on-demand processes, including inkjet or drop-wise deposition methods; (3) extrusion-based technologies like fused deposition modeling (FDM) and direct-ink-write printing; and (4) photo-polymer-based printing techniques like stereolithography (SLA). Numerous polymers have been effectively utilized as source materials in these printing techniques [117]. Three-dimensional-printing polymer composites present both advantages and drawbacks. Depending on the polymer’s structure, state (liquid or solid), and physical properties (viscosity and melting temperature), each process has specific requirements [118]. The range of materials suitable for polymer additive manufacturing in load-bearing applications is relatively narrow. Polymers such as PEEK, UHMWPE, PMMA, PLA, PCL, polypropylene (PP), polyvinyl alcohol (PVA), and polyamide (PA) are commonly utilized in selective laser sintering (SLS) techniques for load-bearing applications [119].

### 4.3. Antibacterial Therapy

Numerous studies have examined the utilization of polymers in antibacterial therapies. Though polymers lack inherent antibacterial properties, they are commonly employed as vector molecules for delivering antibacterial drugs to specific target sites. Notably, chitosan stands out among polymers due to its antibacterial properties. Polymeric nanostructures exhibit bactericidal properties attributed to enhanced drug adsorption, controlled drug release, cytocompatibility, and solubility. Polymers are versatile in forming various structures, including large and small vesicles and nanoparticles [120]. The synthesis of these polymers can involve synthetic polymers, such as polylactic-co-glycolic acid (PLGA) and polylactic acid (PLA), as well as natural polymers like chitosan. Different polymers encapsulate metal and pharmacological nanoparticles endowed with antibacterial characteristics. Chitosan’s inherent antimicrobial properties stem from its positively charged surface. The eradication of bacteria is achieved by altering membrane permeability through electrostatic interactions between positively charged chitosan molecules and negatively charged bacterial membranes. This characteristic has been leveraged in research focused on developing chitosan-based antibacterial nanostructures. For instance, a recent work by Ejaz et al. [121] involved the creation of mannose-modified chitosan nanostructures, demonstrating effective antibacterial activity against both Gram-positive and Gram-negative bacteria. Additionally, Kritchenkov et al. utilized ultrasound-assisted catalyst-free thiol-yne click chemistry to chemically modify chitosan, resulting in a betaine derivative with superior antibacterial properties compared to widely used medications [122]. To combat drug-resistant Gram-positive bacteria, a cationic polymer micelle has also been synthesized [123].

Synthetic polymers such as PLA and PLGA have undergone extensive investigation for efficient drug delivery alongside natural chitosan polymers. Studies have demonstrated their utility as fundamental components for functionalizing ligand molecules to target bacteria. For example, Ucak et al. utilized aptamers against S. aureus to target bacteria to overcome antibiotic resistance. The antibacterial effectiveness of PLGA-based nanoparticles and their ability to transport antibiotics and other potent bactericidal biomolecules have been examined in various studies [124,125]. Da Costa et al. modified the outer surface of PLA nanoparticles with poly-L-lysine to investigate their antibiofilm properties, aiming to disrupt bacterial membranes and enhance the delivery of rifampicin antibiotics [126]. In a separate study, essential oil was encapsulated in PLA nanoparticles for utilization against both Gram-positive and Gram-negative bacteria [127,128].

Microbe-induced infection has a serious negative impact on human life. As a result, several investigations have been conducted to develop novel antimicrobial drugs to combat diseases. New substances with antibacterial characteristics are still being developed. In biomedical applications, polymers having antibacterial and antifungal properties are frequently employed as microorganisms develop resistance to currently available medications. Due to their strong biological reactivity, conducting polymers are attractive in biomedicine [129]. To combat microbial contaminations, polymer-based compounds have been created [130]. To improve the conducting polymer polymer-based composites’ conductivity, photocatalytic activity, and antibacterial activity, a range of nanoparticles, including zinc oxide, cobalt ferrite, and silver, have been added [131,132]. Additionally, biopolymers have been used to improve the composite’s biocompatibility. As a result, nanocomposites made of conducting polymers have been used in biological applications. The ability of biodegradable polymers to spontaneously decompose over a short period in nature is a benefit of employing them. Therefore, it is crucial to coordinate the materials’ transition from a biostable to a biodegradable structure. Using the disc diffusion technique, Sumitha et al. (2012) [133] investigated the antibacterial effectiveness of PCL-silver nanofibrous scaffolds against S. aureus. The absence of antibacterial activity and the zero zone of inhibition, attributed to the lack of silver nanoparticles in the scaffolds, indicated the inefficacy of bare PCL against bacteria. However, the inhibitory zone against Gram-positive bacteria increased as the quantity of silver nanoparticles increased. Cabuk et al. (2014) [134] used a chemical oxidative radical polymerization process to create a copolymer of polypyrrole and chitosan. The composites were identified by performing spectral, thermal, and morphological analyses. In the copolymer chains, there were noticeable strong contacts between PPY and CS, and grafting was shown to boost CS’s electrical conductivity. Comparing PPY and CS to PPY-co-CS, antibacterial activity was considerably increased. Ebrahimiasl et al. (2014) [135] developed conductive bionanocomposite films of Ppy, CS, and Zinc oxide nanoparticles electrochemically deposited on an ITO glass substrate using the potentiostatic technique. ZnO NPs were present in the generated conducting polymer bionanocomposite films, which improved the film’s mechanical and conductivity capabilities. Adding ZnO NPs to the Ppy/ZnO/CS NP composite leads to the formation of a substantially stiffer BNC, as evidenced by the increase in storage modulus compared to the composite without ZnO NPs. This enhancement makes it well suited for applications in drug delivery systems, biosensors, and surgical instruments. Additionally, it demonstrates robust antimicrobial activity, attributed to the electrical characteristics of polypyrrole, the antibacterial and anti-inflammatory properties of zinc oxide, and the biodegradability of chitosan.

Due to the synergistic impact of the fillers, Eren et al. (2015) [136] demonstrated that the combination of carbon nanotubes and silver NPs with conducting polymer, such as PANI, exhibited stronger antibacterial activity compared to PANI-carbon nanotubes and PANI-Ag nanocomposites. Shoja et al. (2015) [137] produced PCL/ZnO composites through the solution-casting technique. The antibacterial properties of the PCL/ZnO microcomposite films were evaluated using the agar disc diffusion method against the Gram-positive Bacillus subtilis and the Gram-negative Salmonella choleraesuis. The PCL/ZnOmicrocomposite antibacterial effects showed that increasing the ZnO content improved the antibacterial activity. Hou et al. (2016) [138] employed an in situ emulsion polymerization process to create nanocomposites containing antibacterial nanoparticles such as zinc oxide and silver. Compared to Ag/ZnO composites, these composites demonstrated synergistic antibacterial properties. Kumar et al. (2016) [139] used a solvent precipitation approach to create biodegradable multifunctional hybrid composites of polycaprolactone reinforced with grapheme/silver nanoparticles. In the PCL/rGO-Ag composite, hMSC proliferation and mineralization were improved without the cytotoxicity of silver nanoparticles. Additionally, the combined effects of Ag and rGO nanoparticles endowed PCL/rGO-Ag with strong antibacterial properties. Thus, rGO-Ag can be a beneficial filler to enhance the multifunctional characteristics of commonly used biodegradable polymers for biomedical applications. According to Shahadat et al. (2017) [140], synthesized composite PANI-PVA (polyvinyl alcohol) films proved effective against S. aureus and E. coli. Though no bacterial activity was seen with native PVA, the composite film showed an almost 100% decrease in E. coli and S. aureus growth. The release of H^+^ from PANI caused the bacterial cell wall to break, which was thought to be the cause of the antibacterial effectiveness of PANI-PVA. According to Ehsan Nazarzadeh Zare et al. (2019) [141], PANI has been combined with biopolymers that contain quaternary ammonium salts, including chitosan, to boost the antibacterial activity while also increasing the biocompatibility of PANI. Quaternized chitosan-graft-PANI injectable hydrogels were employed as biocompatible scaffolds for tissue regeneration. In a study by Masim et al. (2019) [142], it was found that the conducting polymer composite with incorporated ZrO_2_ exhibited a more potent antibacterial effect against S. aureus and E. coli compared to the pure conducting polymer. Abou Hammad et al. (2019) [143] used an in situ polymerization technique to create a biodegradable polyaniline, cellulose, and cobalt ferrite nanocomposite. In addition to Candida albicans as a unicellular fungus, the produced nanocomposites show strong antibacterial action against Bacillus subtilis and Escherichia coli. These nanocomposites were biodegradable and naturally conductive (3.5 × 10^−3^S/cm). Biodegradability decreased with increasing cobalt ferrite concentration in nanocomposites, whereas antibacterial nature increased. Behzad Mohammadi et al. (2019) [144] employed a chemical polymerization technique to create a biodegradable film based on chitosan and polyaniline, exhibiting antifungal, antibacterial, and conductive properties. The study revealed that adding varying concentrations of aniline enhanced the chitosan film’s mechanical, electrical, and antibacterial characteristics. The film demonstrated significant antibacterial efficacy, showcasing the strong antimicrobial properties of the nanocomposites. Muoz-Escobar et al. (2019) [145] found that produced membranes containing PCL and CuO NPs had the strongest antibacterial action against pathogenic bacteria such as S. aureus, E. Coli, and others, considerably reducing the risk of postoperative infections. To develop conductive, biodegradable, and biocompatible hydrogels, Youssef et al, (2019) [146] employed a bionanocomposite composed of chitosan, polyacrylic acid, and polypyrrole loaded with various ratios of Ag-NPs (CS/PAA/PPy/Ag-NPs). This configuration significantly augmented the antibacterial activity of the hydrogel bionanocomposites against various microorganisms. Talebi et al. (2019) [147] innovatively crafted a conductive composite film using the casting method, combining polycaprolactone, chitosan, and polypyrrole (PCL/chitosan/PPy). Comparative analysis with the PCL/chitosan film demonstrated that the PPy-containing films exhibited superior antibacterial properties against E. Coli and S. aureus. Mirmohseni et al. (2019) [148] employed a facile solution polymerization technique to create a PANI-CS-modified TiO_2_ ternary nanocomposite. The development of some bacterial strains can be inhibited by a nanocomposite that has greatly increased conductivity. It might, therefore, be utilized as a powerful antibacterial and antistatic agent. Balitaan et al. (2020) [149] synthesized β-chitin-g-PANI composites through the in situ graft copolymerization of β-chitin with conducting PANI. The study findings demonstrated enhanced antibacterial properties, biodegradability, and conductivity in the composites. The synthesized composite’s distinctive features show their potential for use in the creation of wound care solutions. Hooman Golbaten-Mofrad et al. (2021) [150] fabricated PGS-U/PPy/ZnO scaffolds through salt leaching. Adding PPy increased electrical conductivity, and incorporating ZnO particles improved the scaffolds’ surface hydrophilicity and antibacterial activity. Further research could explore additional enhancements to optimize these nanocomposites for effective electrically conductive scaffolds in tissue-engineering applications. Based on the results obtained, Unnikrishnan et al. (2024) [151] have concluded that the composite with a higher particle concentration emerges as an ideal candidate for future wound-dressing applications. This composite exhibits notable attributes such as electroconductivity, antimicrobial activity, hemostatic ability, and biocompatibility. Hasan et al. (2023) [152] conducted a Density Functional Theory (DFT) study to analyze Natural Bond Orbitals (NBO) for charge and spin distribution, as well as HOMO, LUMO, and molecular electrostatic potential (MEP). The characterization results indicate that the synthesized polymer nanocomposites hold promise for various optoelectronic applications.

## 5. Conclusions

In numerous biomedical applications spanning the past few decades, biodegradable conducting polymers have played roles in tissue engineering, drug delivery, vascular grafts, surgical sutures, and various other applications. This list could be extended to accommodate the needs of increasingly varied and specialized applications. It is challenging to design and manufacture an optimal electroactive polymer that complies with biodegradability and biocompatibility principles, yet it is necessary to lessen the risk that non-degradable particles would trigger an inflammatory response in the host tissue. Although these kinds of polymers have demonstrated biocompatibility in vitro, significant advances have been made in the synthesis and functionalization of these polymer-based biomaterials. However, comprehensive studies on their in vivo biocompatibility and biodegradability are still required. Extensive in vivo experiments examining long-term cytotoxicity and biodegradation are necessary to confirm the nontoxicity and biodegradability of these conductive biomaterials. Another difficult issue arises while synthesizing conducting polymers, because toxic and environmentally hazardous organic solvents break down the biodegradable conducting polymers. In conclusion, electrically conductive polymeric materials are experiencing rapid growth in their potential applications within the electronics and electrical sectors. The necessity for electrically conducting biodegradable polymers has become increasingly urgent in light of recent advancements in environmental sustainability. This review has also investigated biodegradable polymeric systems, highlighting their diverse functionalities that contribute to sustainable and eco-friendly development. Additionally, the review explores the utilization of biodegradable conducting polymers in tissue engineering, biomedical implants, and antibacterial applications. For this reason, the synthesis of hydrophilic and processable materials remains attractive in biological fields. However, this group of innovative biomaterials has developed into an explosive substance that will contribute greatly to biomedical science during the coming decades.

## Figures and Tables

**Table 1:** Various kinds of synthetic polymers and their applications are listed.

S.No.	Polymers	Area of Applications	Ref.
1	Polyethylene glycol/PLGA	Improved oral administration for enhanced cellular absorption and hypoglycemic impact.	[59]
2	PLA (Polylactic acid)	Biomaterials find applications in various fields, including elevated apoptosis and cytotoxicity, barrier membranes, drug delivery, orthopedic applications, guided tissue regeneration (in dental applications), stents, staples, sutures, and tissue engineering.	[60,61]
3	Albumin	Folate-conjugated albumin enhances the potential for activated macrophage cells to reach their target.	[62]
4	Polycaprolactones (PCL)	Biomaterials are crucial in various fields, including orthopedics, guided tissue regeneration in dentistry, Ethicon’s implantable contraception, Monocryl suture, Capronor, barrier membranes, drug delivery, stents, and tissue engineering.	[63,64]
5	Polybutyrate adipate terephthalate (PBAT)	Applications of packaging include the utilization of bottles.	[65]
6	Polyesteramides (PEA)	Hydrogels find applications in drug delivery, tissue engineering, and smart materials, particularly temperature-sensitive ones.	[66]
7	Polyhydroxyvalerate (PHV)	Cons: Limited biodegradability ranging from three to twelve months for products such as Paper Mate, BioTuf, Rubbermaid, Calphalon, agricultural film, dung bags, and client packing materials.	[67]
8	Poly(alkylenealkanoate)s (PBS)	Injection molding is commonly used to produce single-use items like forks and spoons, as well as textiles, fishing equipment, and plant pots.	[68]
9	Thermoplastic starch (TPS)	Applications of packaging	[69]
10	Polycyanoacrylates	Drug delivery and adhesives	[70]
11	Polyanhydrides	Bioactive substance delivery	[71]
12	Poly(amino acids)	Drug delivery, tissue engineering, and orthopedic applications are the areas where these technologies find significant utilization.	[72]
13	Poly(ortho ester)	Drug delivery in the context of stents involves the controlled release or administration of medications directly to the target site where the stent is placed.	[73]
14	Polyphosphazenes	These technologies are crucial in skeletal reconstruction, drug delivery, and blood-contacting devices, among other applications.	[74]
15	Poly(propylene fumarate)	Utilizing medical procedures, equipment, or therapies specifically designed for the musculoskeletal system, consisting of bones, joints, ligaments, tendons, and muscles, is referred to as orthopedic applications.	[75]
16	Polyhydroxybutyrate (PHB)	Biomaterials are crucial in various fields, including orthopedics, guided tissue regeneration in dentistry, barrier membranes, drug delivery, stents, sutures, and tissue engineering.	[76]
17	Polydioxanone	Medical materials and interventions find applications in wound clips, sutures, and fracture fixation in non-load-bearing bones.	[77]
